# Small RNAs in plants: recent development and application for crop improvement

**DOI:** 10.3389/fpls.2015.00208

**Published:** 2015-04-02

**Authors:** Ayushi Kamthan, Abira Chaudhuri, Mohan Kamthan, Asis Datta

**Affiliations:** ^1^National Institute of Plant Genome ResearchNew Delhi, India; ^2^Indian Institute of Toxicology ResearchLucknow, India

**Keywords:** miRNA, siRNA, RNAi, gene silencing, crop improvement

## Abstract

The phenomenon of RNA interference (RNAi) which involves sequence-specific gene regulation by small non-coding RNAs, i.e., small interfering RNA (siRNA) and microRNA (miRNA) has emerged as one of most powerful approaches for crop improvement. RNAi based on siRNA is one of the widely used tools of reverse genetics which aid in revealing gene functions in many species. This technology has been extensively applied to alter the gene expression in plants with an aim to achieve desirable traits. RNAi has been used for enhancing the crop yield and productivity by manipulating the gene involved in biomass, grain yield and enhanced shelf life of fruits and vegetables. It has also been applied for developing resistance against various biotic (bacteria, fungi, viruses, nematodes, insects) and abiotic stresses (drought, salinity, cold, etc.). Nutritional improvements of crops have also been achieved by enriching the crops with essential amino acids, fatty acids, antioxidants and other nutrients beneficial for human health or by reducing allergens or anti-nutrients. microRNAs are key regulators of important plant processes like growth, development, and response to various stresses. In spite of similarity in size (20–24 nt), miRNA differ from siRNA in precursor structures, pathway of biogenesis, and modes of action. This review also highlights the miRNA based genetic modification technology where various miRNAs/artificial miRNAs and their targets can be utilized for improving several desirable plant traits. microRNA based strategies are much efficient than siRNA-based RNAi strategies due to its specificity and less undesirable off target effects. As per the FDA guidelines, small RNA (sRNA) based transgenics are much safer for consumption than those over-expressing proteins. This review thereby summarizes the emerging advances and achievement in the field of sRNAs and its application for crop improvement.

## Introduction

RNA interference (RNAi) is a gene silencing phenomenon that involves sequence-specific gene regulation induced by double stranded RNA (dsRNA) resulting in inhibition of translation or transcription. During the 1990s, post-transcriptional gene silencing (PTGS) was reported in many organisms including fungi, animals, ciliates, and plants (Baulcombe, 2000; Matzke et al., 2001). The phenomenon of gene silencing was discovered accidentally in petunia flowers where expression of chalcone synthase (pigment producing gene), resulted in variegated flowers instead of expected deep purple color. Since, the expression of both the transgene and the homologous endogenous gene was suppressed, the phenomenon was termed co-suppression (Napoli et al., 1990; Campbell, 2005). A similar phenomenon reported in the fungus *Neurospora crassa* was named quelling (Romano and Macino, 1992; Cogoni et al., 1996; Fire et al., 1998), and a related mechanism was identified in animals known as RNAi. The phenomenon of RNAi involves small non-coding RNAs called small interfering RNA (siRNA) and microRNA (miRNA) that are the cleavage product of dsRNA. A ribonuclease called DICER or a Dicer-like enzyme (DCL; Pare and Hobman, 2007) carry out this cleavage. The phenomenon of RNAi, in addition to small non-coding RNAs involves a RNA-induced silencing complex (RISC; Redfern et al., 2013; Wilson and Doudna, 2013) and Argonaute proteins (AGOs; Ender and Meister, 2010; Riley et al., 2012).

In plants, miRNAs were identified about 10 years later after discovery of animal miRNAs. In early, Gunnery and Datta (1987) for the first time reported small inhibitory RNA in plant system involved in translational control. They isolated a small molecular weight RNA from barley embryos that specifically inhibits translation initiation. In mid-2002, small RNAs (sRNAs) with miRNA characteristics were reported by different groups to be present in *Arabidopsis* (Llave et al., 2002b; Mette et al., 2002; Park et al., 2002; Reinhart et al., 2002).

In recent times, RNAi technology has evolved as an important tool of genetic engineering and functional genomics aimed for crop improvement.

## miRNA vs. siRNA

microRNAs (miRNA) and short-interfering RNAs are known to be important regulators of gene expression (Bartel, 2004; Zamore and Haley, 2005; Vazquez, 2006). Though, they show similarity in size (20–24 nt), but differ in precursor structures, pathway of biogenesis, and modes of action (Axtell, 2013; **Table 1**). Both are processed from long RNA precursors by Dicer-like ribonucleases (Lee et al., 1993; Hamilton and Baulcombe, 1999; Hammond et al., 2000; Zamore et al., 2000; Bernstein et al., 2001; Hutvagner et al., 2001). Both regulate the target gene repression (Lee et al., 1993; Hammond et al., 2000; Zamore et al., 2000) through ribonucleoprotein silencing complexes.

**Table 1:** **Comparison of miRNA and siRNA**.

Properties	miRNA	siRNA
Origin	Distinct genomic loci. Encoded by their own genes	Encoded by transposons, viruses, heterochromatin
Biogenesis (nature of precursor)	Single RNA molecules that include an imperfect stem–loop secondary structure	Long bimolecular RNA duplexes or extended hairpins
Evolutionary conservation	Nearly always conserved in related organisms	Rarely conserved in related organisms
Nature of regulatory target	Regulate different genes	Mediate the silencing of the same (or very similar) genes from which they originate

## Short-Interfering RNAs (siRNAs)

Long dsRNA or short-hairpin RNA (shRNA) precursors, which are homologous in sequence to the target gene to be silenced (Fire et al., 1998; Tuschl, 2001), initiate the process of RNAi. The entry of a virus, a genetic element like transposons or an introduced transgene provide long dsRNA can triggers the RNAi pathway. The dicer enzyme is recruited in the cell (Bernstein et al., 2001) leading to the cleavage of the dsRNA into siRNA (Hamilton and Baulcombe, 1999) that are short, 5′-phosphorylated dsRNAs (21–25 nt) with two nucleotide overhangs at the 3′ end (Bernstein et al., 2001; Elbashir et al., 2001). The recruitment of siRNA-induced silencing complex (siRISC) leads to degradation of sense strand of siRNA (having the same sequence as the target gene) The siRISC is then incorporated into the antisense strand of siRNA which in association with AGO and other effector proteins brings about cleavage of the target mRNA in sequence-specific manner. The activated RISC can repeatedly participate in mRNA degradation and protein synthesis inhibition resulting into PTGS.

On the basis of origin and mechanism of biogenesis, sRNAs in plants exhibit a range of size classes, predominantly from 20 to 24 nt. Four Dicer-like nucleases (DCLs) have been reported in *Arabidopsis* (Henderson et al., 2006). Depending on their origin and structure, dsRNA triggers of RNAi are cleaved by one or more DCLs (Zamore et al., 2000; Bernstein et al., 2001). DCL4 and DCL2 are involved in processing of dsRNA with perfectly complementary to produce 20- to 22-nt long short-interfering RNA (Hamilton and Baulcombe, 1999; Hamilton et al., 2002; Hannon, 2002; Tang et al., 2003; Bouche et al., 2006). MiRNAs that are 21-nt long are produced by cleaving of partially paired dsRNA precursors by DCL1 (Denli and Hannon, 2003; Bartel, 2004). DCL3 is involved in biogenesis of 24-nt long repeat-associated sRNAs known as heterochromatic siRNAs (hcsiRNAs; Xie et al., 2005). In a complex called RISC, siRNAs and miRNAs (20- to 21-nt long) in association with AGO proteins, direct sequence-specific cleavage of complementary target RNAs or interfere with their translation (Hammond et al., 2000; Hannon, 2002; Brodersen et al., 2008). Traditionally, sRNA species that are 24-nt long (derived from transposable elements and other repetitive sequences) are linked to transcriptional gene silencing via DNA methylation and repressive chromatin modification (Hamilton et al., 2002; Zilberman et al., 2003; Liu et al., 2004; Fagegaltier et al., 2009; Law and Jacobsen, 2010; Burkhart et al., 2011). SiRNA recruits several DNA- and histone-modifying proteins including the cytosine methyltransferase, chromomethylase3 (Ossowski et al., 2008) which in turn mediate the formation of a transcriptionally inactive silent chromatin state. However, 21 nt long siRNAs may also participate in siRNA-dependent methylation of genomic loci (Pontier et al., 2012). AGO is an important protein that complexes with sRNA to form the core of the RISC. In *Arabidopsis thaliana*, 10 different AGO proteins are known to mediate the effects of several distinct types of sRNAs (Vaucheret, 2008). Endonucleolytic cleavage (slicing) activity catalyzed by the AGO protein (Huntzinger and Izaurralde, 2011) is an important step in post-transcriptional silencing. Guide RNA confers sequence specificity to any RNA silencing reaction whereas the precise nature of silencing is determined by the properties of the associated AGO protein, including its ribonuclease activity, interacting proteins, and subcellular localization.

Movement of plant sRNAs falls into two main categories: cell-to-cell (short-range) and systemic (long-range) movement (Melnyk et al., 2011). Local movement is symplastic, i.e., from the site of initiation to neighboring cells through channels called plasmodesmata (Lough and Lucas, 2006). RNA silencing also spreads systemically over long distances through the vascular phloem tissue. This long distance or systemic movement of the silencing signal takes place over days (Voinnet et al., 1998) and it is generally from photosynthetic sources (i.e., leaves) to sucrose sinks (i.e., roots and growing points) through a bulk flow process. The systemic RNA silencing signal has been identified in plants by direct sampling of phloem sap (Yoo et al., 2004; Buhtz et al., 2008) and detection of RNAs in stocks and scions of grafted plants (Palauqui et al., 1997; Schwach et al., 2005; Brosnan et al., 2007; Dunoyer et al., 2010a; Molnar et al., 2010). Mobile silencing operates in a nucleotide-sequence-specific manner and its components include sRNA molecules (21–24 nt). Molnar et al. (2010) have reported mobile sRNAs of the 24-nt size class that are associated with DNA methylation of targeted loci. It was consistent with the analysis of viral suppressors of systemic silencing in *Nicotiana benthamiana* (Hamilton et al., 2002) and the presence of 24-nt sRNA in the phloem sap of oilseed rape (Buhtz et al., 2008) and pumpkin (Yoo et al., 2004). Dunoyer et al. (2010a,b) showed that the mechanically delivered, fluorescently labeled 21- and 24-nt siRNAs move from cell to cell and over long distances. Artificial miRNAs (amiRNAs) were shown to move short distances in leaves (Schwab et al., 2006) or between the pollen vegetative cell cytoplasm and the sperm cells (Slotkin et al., 2009). Endogenous 21-nt miRNAs (miR399) could also be mobile between shoots and roots (Pant et al., 2008) and within the roots (miR165a and miR166b; Carlsbecker et al., 2010).

RNA interference can be used to achieve desirable traits in crops by manipulating the gene expression (**Table 2**). RNAi mediated gene silencing technique mainly involves identification of the target gene(s) followed by generating RNAi construct with hairpin cassette (gene cloned in sense and antisense orientation flanking a spacer or intron), plant transformation and finally screening and evaluating the traits.

**Table 2:** **Crop improvement by siRNA based RNA interference**.

Traits improvement	Targeted gene	Plant	Reference
**Plant architecture**
Biomass	*OsDWARF4*	Rice	Feldmann (2006)
Grain yield	*OsGA20ox2*	Rice	Qiao et al. (2007)
**Fruit improvement**
Carotenoid and flavonoid	*DET1*	Tomato	Davuluri et al. (2005)
β-Carotene and lycopene	*SlNCED1*	Tomato	Sun et al. (2012)
Carotenoid	Lycopene epsilon cyclase	Rapeseed	Yu et al. (2007)
Decreased Ethylene	ACC oxidase	Tomato	Xiong et al. (2005)
Decreased Ethylene	ACC synthase (*ACS*)	Tomato	Gupta et al. (2013)
Increased shelf life	α-Man/β- Hex	Tomato	Meli et al. (2010)
Seedless fruit	Chalcone synthase	Tomato	Schijlen et al. (2007)
**Nutritional improvement**
Improved stearic- and oleic- fatty acids	Δ9-desaturase and oleoyl- phosphatidylcholine γ6-desaturase	Cotton	Liu et al. (2002)
Low glutenin content	*GluB*	Rice	Kusaba et al. (2003)
Amylose	*SBE IIa and SBE IIb*	Wheat	Regina et al. (2006)
Tearless onion	Lachrymatory factor synthase *(LFS)*	Onion	Eady et al. (2008)
Reduction of toxic terpenoid gossypol	Delta-cadinene synthase	Cotton	Sunilkumar et al. (2006)
**Biotic stress**
** Virus resistance**			
*Bean Golden Mosaic Virus* (BGMV)	*AC1*	Bean	Bonfim et al. (2007)
*Barley Yellow Dwarf Virus* (BYDV)	*BYDV-PAV*	Barley	Wang et al. (2000)
*Rice Dwarf Virus* (RDV)	*PNS12*	Rice	Shimizu et al. (2009)
*Turnip Yellow Mosaic Virus* (TYMV)	*P69*	Tobacco	Niu et al. (2006)
*Turnip Mosaic Virus* (TuMV)	*HC-Pro*	Tobacco	Niu et al. (2006)
**Insect resistance**
*Helicoverpa armigera*	*CYPAE14*	Cotton	Mao et al. (2007)
Corn rootworm	*V-ATPase A*	Maize	Baum et al. (2007)
**Nematode resistance**
*Meloidogyne incognita*	Splicing factor and integrase	Tobacco	Yadav et al. (2006)
*Meloidogyne incognita*	*16D10*	*Arabidopsis*	Huang et al. (2006)
**Bacterial resistance**
* Xanthomonas citri* subsp. citri (Xcc)	*PDS* and *CalS1*	Lemon	Enrique et al. (2011)
*Agrobacterium tumefaciens*	*iaaM* and *ipt*	*Arabidopsis*	Escobar et al. (2001), Dunoyer et al. (2006)
**Fungal resistance**
*Magnaporthe grisea Xanthomonas oryzae*	*OsSSI2*	Rice	Jiang et al. (2009)
*Magnaporthe grisea*	*OsFAD7* and *OsFAD8*	Rice	Yara et al. (2007)
*Phytophthora infestans*	*SYR1*	Potato	Eschen-Lippold et al. (2012)
*Blumeria graminis* f. sp. tritici	*MLO*	Wheat	Riechen (2007)
**Enhanced drought tolerance**
	Farnesyl transferase	Canola	Wang et al. (2009)
	C-kinase 1 (*RACK1*)	Rice	Li et al. (2009)
	*OsDSG1*	Rice	Park et al. (2010)
	*OsDIS1*	Rice	Wang et al. (2011)

## microRNA (miRNA)

Plant miRNAs are a class of small regulatory RNAs (20–22 nt) that are encoded by endogenous miRNA genes and transcribed by RNA polymerase II into primary miRNAs (pri-miRNA) having partially double- stranded stem–loop structures (Jones-Rhoades et al., 2006). Following transcription, pri-miRNA is cleaved by a DCL1 enzyme in a two step process resulting in production of a pre-miRNA and a mature miRNA duplex (miRNA/miRNA^∗^). The mature miRNA is incorporated into RISC and mediates the degradation of mRNA target. miRNA can down-regulate the level of protein of their target genes through either translational repression (Aukerman and Sakai, 2003; Chen, 2004; Brodersen et al., 2008), through cleavage of transcript (Llave et al., 2002a; Xie et al., 2003), or transcriptional inhibition (Bao et al., 2004; Khraiwesh et al., 2010).

In animals, miRNA mostly act by translational inhibition, where they often bind motifs in the 3′ UTRs of their targets, which show several mismatches to the miRNA. However, in plants target motifs of miRNA have few mismatches which are most often found in the coding sequences and act mostly by transcript cleavage through a mechanism closely related to RNAi. In animals, miRNA usually consist of 20–22 nucleotides where as in plants it is 20–24 nucleotides (Reinhart et al., 2002; Bartel, 2004). Plant miRNAs are less conserved compared to animal miRNAs. In plants, mostly the mature miRNAs are conserved where as miRNA precursors are usually conserved in animals (Bartel, 2004). Similar to animlas, in *Arabidopsis*, a RNAse III-like enzymes carry out processing of miRNAs from primary precursors followed by incorporation into a protein complex named RISC. However, an additional step is included in the biogenesis of plant miRNAs, i.e., the methylation on the ribose of the last nucleotide of miRNA by the methyltransferase Hen1 (Park et al., 2002). Unlike animals, plant miRNAs show high degree of sequence complementarity to their target mRNAs (Rhoades et al., 2002). Because of this fact, bioinformatics prediction of plant miRNA targets is much easier and has facilitated prediction and subsequent validation of many plant miRNA targets.

A majority of plant miRNA targets includes genes encoding for transcription factors (Rhoades et al., 2002; Mitsuda and Ohme-Takagi, 2009), few code for hormone receptors (Navarro et al., 2006), some encode enzymes (Xie et al., 2003; Fujii et al., 2005). Many plant miRNAs are known to regulate post-transcriptional gene expression and play critical roles in many developmental processes, abiotic stresses and pathogen responses (Xin et al., 2010), in nutrient homeostasis etc. Functional characterization of several plant miRNA and their target need high-throughput sequencing at global genome-level (Wang et al., 2011). MiRNAs have been extensively studied in model plants such as rice, *A. thaliana* etc.

Schwab et al. (2006) have designed a series of amiRNAs targeting different endogenous mRNAs and compared their effects to those of natural miRNAs. amiRNAs can be generated by replacing the sequences of miRNA/miRNA*within the miRNA precursor without disrupting its structural features. Like natural miRNAs, amiRNAs had varying number of target mismatches and could silence both single and multiple target genes with high specificity as determined by genome-wide expression profiling. The direct targets of amiRNAs can be accurately predicted by parameters of target selection already determined for natural miRNAs (Schwab et al., 2005).

Thus, extensive base pairing with targets is required for plant miRNA function. amiRNAs can be efficiently used for gene silencing, especially when there is need to down regulate several related, but not identical, target gene. Transgenic plants harboring amiRNAs under constitutive and inducible promoters have shown specific and efficient down-regulation of target genes of interest with limited non-autonomous effect. Thus, amiRNAs have great potential for crop improvement (**Table 3**).

**Table 3:** **Crop improvement by miRNA based RNA interference**.

Trait improved	microRNA	Target	Plant	Reference
Drought tolerance	miR169	*NFYA5*	*Arabidopsis*	Li et al. (2008b)
	miR169	*GmNFYA3*	Soybean and *Arabidopsis*	Ni et al. (2013)
Cold tolerance	miR319	*PCF5/PCF8*	Rice	Yang et al. (2013)
Heat stress tolerance	miR398	*CSD1 CSD2 CCS*	*Arabidopsis*	Guan et al. (2013)
Grain size, shape, and quality	miR156	*SPL14/SPL16*	Rice	Jiao et al. (2010), Miura et al. (2010), Wang et al. (2012)
	miR397	*OsLAC*	Rice	Zhang et al. (2013)
Parthenocarpy	miR167	*ARF8*	*Arabidopsis* and tomato	Goetz et al. (2007)
Bacterial resistance	miR393	*TIR1*	*Arabidopsis*	Navarro et al. (2006)
*Cauliflower mosaic virus* (CMV) resistance	amiR171	2b of CMV	Tobacco	Qu et al. (2007).
*Tomato leaf curl New Delhi virus* (ToLCNDV) resistance	amiR-AV1-1	AV1/AV2	Tomato	Vu et al. (2013)
*Turnip Yellow Mosaic Virus* (TYMV) and *Turnip Mosaic Virus* (TuMV) resistance	amiR159	P69 of TYMV and HC-Pro of TuMV	*Arabidopsis*	Niu et al. (2006)

## RNAi and its Role in Crop Improvement

### Plant Architecture

#### Biomass

RNA interference can be employed successfully to improve yield of crop and fruit plants by manipulating the basic agronomic traits of plant such as height, inflorescence, branching and size. RNAi mediated knockdown of a gene *OsDWARF4* in rice resulted in shorter plants with erect leaf architecture leading to increased photosynthesis in the lower leaves. Such plant has potential for improved yields under dense planting conditions (Feldmann, 2006).

Some plant materials show recalcitrance to a process of saccharification which causes major limitation for conversion of lignocellulosic biomass to ethanol. The problem of recalcitrance of plant cell wall to bioconversion can be overcome by genetic reduction of lignin content (Chen and Dixon, 2007). Independent down-regulation of each of six lignin biosynthesis enzymes in transgenic alfalfa lines, yielded nearly twice as much sugar from stem’s cell walls as compared to wild-type plants (Reddy et al., 2005). Down-regulation of lignin genes like cinnamate 4-hydroxylase (C3H), shikimate hydroxycinnamoyl transferase (HCT) and 4-coumarate-coA ligase (4CL) in plants reduced total lignin content, improved accessibility of cellulases for cellulose degradation and increased dry matter degradability (Hisano et al., 2009). This lignin modification also facilitated bypassing the need for acid pretreatment (Chen and Dixon, 2007). Over-expression of the maize Corngrass1 (Cg1) miRNA that belongs to the MIR156 family caused prolonged vegetative phase and delayed flowering (Chuck et al., 2011) resulting in increased biomass. The transgenic plants showed up to 250% more starch and improved digestibility (Chuck et al., 2011). It was reported that the degrees of morphological changes and biomass yields were related to the expression level of an exogenous rice miRNA Osa-miR156b (Fu et al., 2012). Transgenic switch grass plants with relatively high levels of miR156 showed highly stunted growth, whereas those with moderate and low levels of miR156 expression had 58–101% more biomass production than wild-type controls as a result of increases in tiller numbers. It was also observed that over-expression of rice miR156 could improve the yield of solubilised sugar as well as forage digestibility. Over-expression of miR156 led to increased biomass in other plant species including *Arabidopsis* and rice (Schwab et al., 2005; Xie et al., 2006, 2012). Cg1 over-expressing, poplar transgenics plants showed significant increase in the growth of axillary meristem, shorterning of internode length, and reduction in stem lignin content (up to 30%) compared to that of the wild-type (Rubinelli et al., 2013).

#### Grain Yield

Plant architecture can be manipulated in order to achieve increased grain yield like in rice plants (Jiao et al., 2010; Miura et al., 2010; Springer, 2010; Wang et al., 2012). RNAi-mediated suppression of GA 20-oxidase (*OsGA20ox2*) gene resulted in semi-dwarf plants from a taller rice variety QX1. This RNAi transgenic exhibited significant increase in panicle length, increased number of seeds per panicle and higher test (1000 grain) weight (Qiao et al., 2007). OsSPL14 (Souamosa promoter binding protein-like 14) was reported to be the target of Osa-miR156 in rice (Jiao et al., 2010; Miura et al., 2010) and was shown to positively regulate the rice plant architecture leading to enhanced yield of rice grain (Jiao et al., 2010). Higher expression of OsSPL14 could modify the rice plant architecture resulting in decreased tiller number and increased grain yield (Jiao et al., 2010; Miura et al., 2010; Wang et al., 2012). Wang et al. (2012) showed OsSPL16 to be a positive regulator of cell proliferation with increase in grain width and yield in over-expressing rice plants. However, elevated expression of OsSPL16 resulted in decrease grain appearance quality. This was expected as grain quality and yield are usually negatively correlated. But, decreased expression of OsSPL16 resulted in slender grains with better quality. Thus, miR156 and its targets OsSPL14 or OsSPL16 can be used for modifying plant architecture to create superior rice cultivars with greater yield. Recently, Guo et al. (2013) showed interactions of OsmiR444a regulated OsMADS57, OsTB1 (TEO-SINTE BRANCHED1) and D14 (Dwarf14) in controlling rice tillering. Thus, manupulating OsmiR444a and its targets by genetic engineering can prove to be an important strategy to achieve high grain yield (Guo et al., 2013). Zhang et al. (2013) reported that over-expression of OsmiR397, which is expressed naturally in young panicles and grains of rice promotes panicle branching and enlargement of grain size, causing an increase in overall grain yield of up to 25% in a field trial. OsmiR397 increases grain yield by down-regulating its target, *OsLAC* coding for a laccase-like protein that is involved in the sensitivity of plants to brassinosteroids. Since, miR397 is highly conserved across different species; this strategy can be extended to other cereal crops for increasing grain yield.

### Fruit Improvement

#### Enhanced Nutritional Value and Edibility

Tomato (*Lycopersicon esculentum*) is one of the most economically important fruit crop across the world rich in antioxidants, minerals, fibers, and vitamins (Rajam et al., 2007). RNAi has been utilized in development of tomato fruit with enhanced level of carotenoids and flavonoids which are highly beneficial for human health. RNAi in combination with fruit specific promoter has been used to suppress an endogenous *DET1* gene in tomato, a photomorphogenesis regulatory gene involved in repression of several light controlled signaling pathways (Davuluri et al., 2005). *DET1* was specifically degraded in transgenic tomatoes with suppressed *DET1*, accompanied by an increase in the level of flavonoid and carotenoid. Abscisic acid (ABA) plays important roles during tomato fruit ripening. The SlNCED1 gene of tomato encoding 9-*cis*-epoxycarotenoid dioxygenase), an important enzyme in the ABA biosynthesis, was suppressed by RNAi. The fruits from RNAi lines showed enhanced accumulation of upstream compounds in the pathway, chiefly lycopene and β carotene. Similarly, RNAi has been utilized to increase the carotenoid content of rapeseed (*Brassica napus*) by down-regulating the expression of lycopene epsilon cyclase (*ε-CYC*) gene. The seeds obtained from transgenic *Brassica* showed increased in zeaxanthin, violaxanthin, β-carotene, and lutein content (Yu et al., 2007). RNAi approach has also been used in apple to improve the fruit quality by enhanced self-life (Dandekar et al., 2004), reducing the amount of a major apple allergen (Gilisen et al., 2005) and silencing the leaf sorbitol synthesis which affects fruit quality such as starch accumulation and sugar-acid balance (Teo et al., 2006).

#### Enhanced Shelf Life

The post-harvest deterioration and spoilage of vegetables and fruits are major causes of economic loss. Therefore, increase in shelf life of vegetables and fruits by delayed ripening are another essential agronomic trait that is being addressed through RNAi/microRNA technology. Initiation of ripening in climacteric fruits like tomato is characterized by a climacteric burst of ethylene in, resulting in the regulation of the expression of ripening-specific genes (Osorio et al., 2011). Thus, delayed ripening can be achieved in climacteric fruits by manipulating the gene involved in ethylene production and perception by employing either sense or antisense technology (Hamilton et al., 1990; Oeller et al., 1991; Theologis et al., 1993; Ye et al., 1996; Wilkinson et al., 1997; Xiong et al., 2003, 2005). However, RNAi was shown to be more effective and specific in nature than strategies involving either sense or an antisense RNA (Fire et al., 1998). Xiong et al. (2005) introduced dsRNA targeting a single unit of 1-aminocyclopropane-1-carboxylate (ACC) oxidase, a gene of ethylene biosynthesis pathway in tomato and suppressed the expression of its gene. The rate of ethylene production was significantly inhibited in the ripened fruits of transgenic plants leading to prolonged shelf life. However, a RNAi strategy targeting suppression of more than one homolog would be certainly much effective than knockdown of a single homolog. Gupta et al. (2013) used RNAi technology to develop tomatoes with delayed ripening by targeting three homologs of 1-aminocyclopropane-1-carboxylate (ACC) synthase (ACS) gene during the course of ripening. Delayed ripening leading to extended shelf life for ≈45 days was achieved by effective repression of ethylene production in tomato fruits by using chimeric RNAi-ACS construct designed to target ACS homologs. Two ripening-specific ethylene induced *N*-glycoprotein modifying enzymes, α-mannosidase (α-Man) and β-D-*N*-acetylhexosaminidase (β-Hex) were identified and targeted by Meli et al. (2010) through RNAi technique. Enhanced shelf life of ≈30 days and ≈2–2.5 fold firmer fruits were observed after texture analysis of these transgenic tomatoes as rate of fruit softening was significantly reduced.

microRNAs are involved in tomato fruit development and ripening (Pilcher et al., 2007; Itaya et al., 2008; Moxon et al., 2008; Zhang et al., 2011b; Molesini et al., 2012; Karlova et al., 2013). MiR156 targets a important gene in fruit ripening, colorless never ripe (CNR; Moxon et al., 2008; Molesini et al., 2012). Transgenic tomato plants over-expressing sly-miR156 exhibited slightly lighter color than wild-type controls, but could completely ripen eventually (just later; Zhang et al., 2011b). Furthermore, over-expression of miR156 in tomato also resulted in smaller fruit, increased leaf numbers, but reduced height and decreased leaf size (Zhang et al., 2011b), suggesting that higher fruit yield and shorter ripening time in tomato could be expected through either reducing miR156 expression or releasing one or several miR156 target genes. This needs to be tested by transgenic studies. The most recent study shows that *CNR* is also negatively regulated by *APETALA2a* (*AP2a*), a target of miR172, which positively regulates fruit ripening and negatively regulates ethylene synthesis. At the same time, *CNR* positively regulates *AP2a*, suggesting a feedback loop regulation of fruit development and ripening (Karlova et al., 2013).

#### Seedless Fruit Development (Parthenocarpy)

Parthenocarpy or seedlessness is a process involving production of seedless fruits, developed from the ovary without pollination and fertilization. Parthenocarpy or seedlessness is a highly desirable agronomic trait for fruit which enhances its marketing values (Molesini et al., 2012) because high yield can be achieved even under environmental conditions unfavorable for pollination and fertilization. In tomato, seedless fruits have been achieved by down-regulating a chalcone synthase, a gene involved in first step of flavonoid biosynthesis (Schijlen et al., 2007). Phytohormones such as auxin and gibberellins are closely associated with the trait of parthenocarpy (Molesini et al., 2012) which in turn are regulated by many miRNAs. Thus, manipulating the level of phytohormones by controlling activities of miRNAs or their targets can prove to be an important approach to achieve parthenocarpy. For example, expression of an aberrant form of auxin response factor 8 (ARF8), a target of miR167 (Ru et al., 2006; Wu et al., 2006; Molesini et al., 2012), resulted in parthenocarpic fruit in both *Arabidopsis* and tomato (Goetz et al., 2007). Seedless fruits were observed in tomato plants when *ARF7* function was suppressed using RNAi (De Jong et al., 2009). Parthenocarpic fruits were also observed in tomato in which genes of the AUCSIA family coding for 53-amino-acid-long (protein or peptide) were functionally suppressed by RNAi (Molesini et al., 2009).

### Biotic Stress Resistance

Phytopathogens are the cause many plant diseases leading to substantial damage to crop production and thereby heavy economic loss. Several RNAi strategies have been employed to improve the defense mechanism in crop plants against various biotic stresses including attack by virus, bacteria, fungi, nematodes, and insects.

#### Virus Resistance

Virus-induced gene silencing (VIGS) is a RNA-mediated PTGS mechanism that protects plants against foreign gene invasion (Beclin et al., 2002; Ding, 2010). VIGS has emerged as an extremely powerful functional genomics tool for knocking out gene expression of target plant genes in some plants. The concept of pathogen derived resistance (PDR) has promoted research aimed at achieving plants resistant to virus through genetic engineering (Simon-Mateo and García, 2011). PDR is either protein mediated involving protein encoded by the transgene or RNA-mediated, i.e., by the transcript produced from the transgene. In order to achieve PDR, hairpin dsRNA including small hairpin RNA (shRNA), self-complementary hpRNA, and intron-spliced hpRNA were formed *in vivo* using inverse repeat sequences from viral genomes. Among these, PTGS with the highest efficiency was elicited by the method involving self-complementary hairpin RNAs separated by an intron (Smith et al., 2000; Wesley et al., 2001). High resistance against viruses has been observed in plants even in the presence of inverted repeats of dsRNA-induced PTGS (IR-PTGS; Beclin et al., 2002; Pandolfini et al., 2003; Zrachya et al., 2007). Resistance to *Potato Spindle Tuber Viroid* (PSTVd) infection was achieved in transgenic tomato plants producing dsRNA against PSTVd sequences (Nora et al., 2009). Similar strategy was used to successfully engineer resistance in cassava plants against *African Cassava Mosaic Virus* (ACMV; Vanderschuren et al., 2009). Virus resistance has been engineered successfully in plants by targeting the coat protein (CP) gene through RNAi. Powell-Abel et al. (1986) showed that transgenic tobacco expressing the CP gene of *Tobacco Mosaic Virus* (TMV) was resistant to TMV and that the resistance was due to the expressed CP. Later, this strategy was extended to generate resistance against many different viruses as potato resistant to *Potato Virus Y* (PVY; Missiou et al., 2004), tobacco resistant to *Beet Necrotic Yellow Vein Virus* (BNYVV; Andika et al., 2005), *Cucumis* cv. *melo* resistant to *Papaya Ring Spot Virus* type W(PRSV-W; Krubphachaya et al., 2007), *N. benthamiana* resistant to* Cucumber Green Mottle Mosaic Virus* (CGMMV; Kamachi et al., 2007), *N. benthamiana* and *Prunus domestica* resistant to *Plum Pox virus* (PPV; Hily et al., 2007).

The use of RNA silencing strategy to engineer resistance is not limited to RNA viruses but can successfully be applied to DNA viruses. For example blackgram plants recovered efficiently from geminivirus *Vigna mungo yellow mosaic virus* (VMYMV) infection when inoculated with hpRNA construct containing the promoter sequence of VMYMV under the control of the 35S promoter (Pooggin et al., 2003). RNAi method has been used to generate common bean resistant to geminivirus *Beans Golden Mosaic Virus* (BGMV; Bonfim et al., 2007). A broad-spectrum resistance has been developed against tospoviruses in tomato plants by targeting multiple regions of a viral gene (Bucher et al., 2006). Zhou et al. (2012) have used sequences from disease-specific protein gene and CP gene from *Rice Stripe Virus* to develop resistance against Rice Stripe Disease.

Many viruses express viral silencing repressors (VSRs) proteins to counter host antiviral RNA silencing (Burgyan and Havelda, 2011). One of the strategies to improve virus resistance involves targeting the miRNA against these VSR. Niu et al. (2006) developed *Arabidopsis* plants with specific resistance against *Turnip Yellow Mosaic Virus* (TYMV) and *Turnip Mosaic Virus* (TuMV) by expressing amiRNAs based on miR159 precursor of *A. thaliana*. These amiRNAs target the sequence of two VSRs, P69 of TYMV and HC-Pro of TuMV. Transgenic tobacco resistant to *Cucumber Mosaic Virus* (CMV) was generated by targeting a VSR, 2b of CMV through expression of an amiRNA based on an *A. thaliana* miR171 precursor (Qu et al., 2007). TGBp1/p25 of *Potato Virus X* (PVX) was targeted in tobacco by expression of amiRNAs based on an *A. thaliana* miR159a, miR167b, and miR171a precursors (Ai et al., 2011). Singh et al. (2014) generated transgenic tobacco plants resistant to *Tomato leaf curl New Delhi virus* (ToLCNDV) by transformation with *trans*-acting siRNA generating constructs against RNAi suppressor proteins (AC2 and AC4) of the geminivirus. Another promising strategy to reduce the multiplication and spread of virus in the plant includes the use of amiRNAs targeting the viral genes involved in replication, transmission, and symptom development after viral infection. Vu et al. (2013) used two amiRNA targeting the middle region of the AV1 (coat protein) transcript (amiR-AV1-3) and the overlapping region of the AV1 and AV2 (pre-coat protein) transcripts (amiR-AV1-1) of a geminivirus, *Tomato leaf curl virus* (ToLCV). Transgenic tomato plants expressing amiR-AV1-1, were highly tolerant to ToLCNDV and could successfully propagate until the T2 generation.

#### Bacterial Resistance

Bacterial diseases are extremely difficult to control due to rapid rate of spreading. RNAi mediated supression of two genes of *Agrobacterium tumefaciens* involved in crown gall tumor formation (iaaM and ipt) could significantly reduce the production of tumors in *Arabidopsis* (Escobar et al., 2001; Dunoyer et al., 2006). This strategy could be further extended to other plants. Fatty acids and their derivatives are important signaling molecule reported to negatively regulate plant’s resistance to bacterial disease (Li et al., 2008a; Jiang et al., 2009). *Arabidopsis* and soybean plants showing enhanced resistance to multiple pathogens were generated by RNAi mediated suppression of *SACPD* gene encoding a fatty acid desaturase (Jiang et al., 2009).

In *Arabidopsis*, miR393 was reported to repress auxin signaling by negatively regulating the F-box auxin receptors like *TIR1*, thereby restricting the infection by bacteria *Pseudomonas syringae* (Navarro et al., 2006). Transgenic *Arabidopsis* plants over-expressing miR393 had enhanced bacterial resistance but with some developmental alterations (Navarro et al., 2006). Two different miRNAs, miR398 (Jagadeeswaran et al., 2009) and miR825 (Fahlgren et al., 2007) were reported to be down-regulated by bacterial infections. In the case of miR398, the expression of miR398 targets coding for two Cu/Zn superoxide dismutases (CSD1 and CSD2) was analyzed, and CSD1 was up-regulated upon bacterial infection in accordance with the down-regulation of miR398 under biotic stress (Jagadeeswaran et al., 2009). Very recently, new insight into miRNA function was gained with the discovery, that several miRNA families target genes of plant innate immune receptors (NBS-LRR) in Legumes (Zhai et al., 2011) and Solanaceae (Li et al., 2012). MiR482/2118 family of miRNAs were shown to target numerous NBS-LRR mRNAs encoding disease resistance proteins in tomato (*Solanum lycopersicum*) and other members of the Solanaceae (Shivaprasad et al., 2012). Viral and bacterial infection suppresses miR482-mediated silencing of *R* genes. Thus, these pathogen responsive miRNA are either up- or down-regulated in response to bacterial invasion and effect gene expression by suppressing negative regulators and inducing positive regulators of immune responses. Identification and characterization of the targets of these miRNAs would help decipher new players in the pathways of host defense. If these pathogen-regulated miRNAs serve as positive regulator of bacterial resistance, strategy to generate transgenics with enhanced bacterial resistance involves constitutive over-expression of miRNA or amiRNA. When miRNAs act as negative regulators, transgenic plants over-expressing these miRNAs become more sensitive to bacteria. In these cases, up-regulation of their target genes might be an effective strategy for improving plant stress tolerance, which can be achieved by over-expressing a miRNA-resistant form of its target or using amiRNA target mimic (Franco-Zorrilla et al., 2007).

#### Fungal Resistance

Targeting the genes of fatty acid metabolism through RNAi has proved to be a important strategy to generate diseases tolerance in various crop plants. RNAi-mediated suppression of a rice gene *OsSSI2* led to enhanced resistance to blast fungus *Magnaporthe grisea* and leaf blight bacterium *Xanthomonas oryzae* (Jiang et al., 2009). Besides, enhanced disease resistance against *M. grisea* in rice was achieved by suppression of two genes namely *OsFAD7* and *OsFAD8* which are Ω-3 fatty acid desaturase (Yara et al., 2007). RNAi mediated targeting of genes involved in lignin production, led to enhanced resistance of soybean to phytopathogen *Sclerotinia sclerotiorum* due to reduced lignin content (Peltier et al., 2009).

Very recently, 24 miRNAs were shown to be involved in responses to attack by the fungus *Blumeria graminis* f. sp. tritici (Bgt) in wheat which cause a devastating diseases of wheat-powdery mildew (Xin et al., 2010). A rice miRNA osa-miR7695 was found to negatively regulate a natural resistance-associated macrophage protein 6 (OsNramp6) in response to the blast fungus *Magnaporthe oryzae*. Improved resistance to rice blast infection was achieved by over-expression of Osa-miR7696 (Campo et al., 2013).

#### Insect and Nematode Resistance

RNA interference has also been applied to control insect pests which lead to substantial crop loss (Huvenne and Smagghe, 2010). The development of a new generation of insect-resistant crops involves feeding of dsRNA as a diet component to insect which was shown to efficiently down-regulate the targeted genes in insect (Price and Gatehouse, 2008). The strategy was used in corn plants by expression of dsRNAs for tubulin or vacuolar ATPase genes to develop western corn rootworm (WCR) resistant transgenic corn plants (Baum et al., 2007). Cotton bollworm (*Helicoverpa armigera*) larvae showed reduced growth when fed on plant material expressing dsRNA specific to cytochrome P450 gene (*CYP6AE14*; Mao et al., 2007). Silencing of genes involved in parasitism or housekeeping genes in the root-knot nematode by expression of dsRNA in host plant resulted in enhanced resistance to the nematode (Gheysen and Vanholme, 2007). Sindhu et al. (2009) has targeted four genes involved in parasitism of sugar beet cyst nematode (*Heterodera schachtii*), having host *A. thaliana* by RNAi. Though complete resistance was not achieved but the number of mature nematode females in different RNAi lines were reduced to 23–64%. Ibrahim et al. (2011) could successfully reduce the gall formation by *Meloidogyne incognita* in soybean roots by suppressing the genes encoding tyrosine phosphatase (TP) and mitochondrial stress-70 protein precursor (MSP).

microRNAs were also reported to be involved in plant nematode interactions. In response to infection by the nematode, *H. schachtii*, miR161, miR164, miR167a, miR172c, miR396c, miR396a,b, and miR398a were down-regulated in *Arabidopsis* (Hewezi et al., 2008; Khraiwesh et al., 2012). Comparative analysis of miRNA profiling in soybean indicated that 101 miRNAs belonging to 40 families were responsive to the infection of the soybean cyst nematode (SCN; *Heterodera glycines*), the most devastating pathogen in soybean. It was further revealed that 20 miRNAs were differentially expressed between SCN resistant and susceptible soybean cultivars (Li et al., 2012). Moreover, it has been suggested that nematode induced miRNAs and sRNAs likely to be involved in feeding site establishment and parasitism, respectively (Hewezi et al., 2008). Over-expression of these nematode induced candidate miRNAs, and/or silencing of their corresponding targets, may provide clear insight about plant-nematode parasitism, and also may provide nematode resistance to crop plants. Nematode resistance can also be achieved by expressing amiRNA, containing known miRNA genes with the replaced seed region of the vital gene (parasitism or housekeeping) of plant parasitic nematodes.

### Abiotic Stress Tolerance

#### Drought and Salinity Tolerance

Water deficit or drought is one the major environmental stresses limiting crop productivity. RNAi has been applied successfully to develop drought tolerant crops. RNAi mediated down-regulation of farnesyl transferase in canola using the *AtHPR1* promoter showed more resistance to seed abortion during flowering induced by water deficiency without affecting yield during drought stress (Wang et al., 2009). Transgenic rice plants tolerant to drought stress were developed by silencing of receptor for activated C-kinase 1 (*RACK1*; Li et al., 2009). The ubiquitin ligase gene has been targeted for RNAi in rice to enhance drought tolerance. Rice knockdown of a RING finger E3 ligase gene-*OsDSG1* leads to enhanced drought tolerance (Park et al., 2010). Similarly, silencing of *OsDIS1* (for *Oryza sativa* drought-induced SINA protein 1), a C3HC4 RING finger E3 ligase by RNAi enhanced drought tolerance.

Wang et al. (2011) studied legume model plant, *Medicago truncatula* and identified several drought-responsive miRNAs. The already known and predicted targets of these miRNAs were found to be involved in wide variety of processes in plant cell including development, transcription, protein degradation, detoxification, etc. It was observed that miR393 was strongly up-regulated by dehydration, cold, high salinity, and ABA treatments (Sunkar and Zhu, 2004). On exposing *Arabidopsis* to varying degrees of different abiotic stress, up regulation was observed in miR402, miR319c, miR389a, and miR397b. Xin et al. (2010) identified and reported 12 miRNAs responsive to heat stress in wheat (*Triticum aestivum* L.). MiRNA responsive to various stresses (drought, cold, salinity, and ABA) were identified in rice seedlings (Jian et al., 2010). Among many stress responsive miRNAs discovered in rice, only two miR393 and miR169 were found to be responsive to abiotic stress like dehydration (Zhao et al., 2007). In *Arabidopsis*, miR169 was down-regulated by drought stress, and its target nuclear factor YA5 (NF-YA5) was significantly induced upon drought stress. Transgenic plants over-expressing miR169a were more sensitive to drought stress with increased leaf water loss as compared to wild-type controls, and over-expression of NF-YA5 led to enhanced drought tolerance in transgenic plants (Li et al., 2008a). Similarly, a newly characterized Soybean (*Glycine max* L.) gene *GmNFYA3*, a target of miR169, was demonstrated to positively modulate drought stress tolerance in transgenic *Arabidopsis* plants (Ni et al., 2013). However, over-expression of *GmNFYA3* in *Arabidopsis* resulted in increased sensitivity to salinity stress and exogenous ABA (Ni et al., 2013). These findings were contrary to the observation in tomato (*S. lycopersicum*), in which miR169 was induced by drought stress and its four targets NF-YA1/2/3 and multidrug resistance-associated protein gene 1 (*MRP1*) were all down-regulated by drought stress. Constitutive over-expression of tomato miR169c led to reduced stomatal openings, transpiration rate, and leaf water loss, thus enhanced drought tolerance in transgenic plants in comparison to wild-type controls (Zhang et al., 2011a). It is notable that transgenic tomato plants had no noticeable morphological and developmental alterations under field conditions, indicating that miR169 or its targets could be potential candidate genes for genetic engineering to achieve enhanced abiotic stress tolerance in transgenic plants (Zhang et al., 2011a). However, as mentioned above, miR169 positively regulates plant response to drought stress in tomato, but negatively in *Arabidopsis*. The opposite roles miR169 play in different plant species suggest that even conserved miRNAs might function in a species-specific manner and this should be taken into careful consideration when tailoring GM strategies for specific target species.

In a recent study, transgenic creeping bentgrass (*Agrostis stolonifera*) plants over-expressing a rice miR319 gene (Osa-miR319) exhibited enhanced tolerance to drought and salinity that was associated with increased leaf wax content and water retention but reduced sodium uptake. Gene expression analysis indicated that the enhanced abiotic stress tolerance can be attributed to a significant down-regulation of at least four putative turf miR319 target genes teosinte branched/cycloidea/proliferating factors (TCP)—AsPCF5, AsPCF6, AsPCF8, and AsTCP14 and a homolog of a rice NAC domain gene AsNAC60 (Zhou et al., 2013). It is noticeable that over-expression of Osa-miR319 caused pleiotropic phenotypes including increased leaf size, enlarged stems and decreased tiller number, which are undesirable traits for creeping bent grass. To better utilize the miR319-based GM strategy, further functional characterization of miR319 targets or downstream genes in the related biological pathways needs to be undertaken. However, the mechanism of miR319-mediated plant abiotic stress response revealed from this study provides important information to develop novel strategies for plant genetic engineering and has the potential to be applied in other important crop species. MiR159 was reported to respond to hormone signaling and dehydration responses in *Arabidopsis* (Achard et al., 2004; Reyes and Chua, 2007).

Hu et al. (2009) showed that most of the rice histone deacetylases genes are responsive to drought or salt stresses with specific patterns of expression and divergent developmental functions compared to closely related homologs in *Arabidopsis*. Trindade et al. (2010) identified several conserved miRNAs in *M. truncatula* plants which are expressed differentially in water-deficit condition. MiR169 was shown to be down-regulated in roots, whereas miR398a/b and miR408 showed very high expression in shoots as well as roots. Hwang et al. (2011) reported a drought stress-responsive miR171 family in potato plants, *Solanum tuberosum* which miR171a, miR171b, and miR171c. The RNAi-mediated silencing of farnesyl transferase genes (*FTA* or *FTB*) in canola resulted in reduced transpiration rate due to decreased stomatal conductance, thereby promoting yield (Wang et al., 2005, 2009).

#### Cold and Heat Stress Tolerance

The expression of miR319 was reported to change in response to cold stress in *Arabidopsis* (Sunkar and Zhu, 2004; Liu et al., 2008), rice (Lv et al., 2010), and sugarcane (Thiebaut et al., 2011). Further transgenic studies using wild-type plants as controls indicated that over-expression of Osa-miR319 gene led to increased cold stress tolerance (4∘C) after chilling acclimation(12^∘^C) of plants (Yang et al., 2013). However, miR319 transgenic rice plants displayed severe developmental delay. To avoid the pleiotropic effect of miR319, two RNAi lines for the miR319 targets, OsPCF5 and OsTCP21, were generated. These RNAi lines, upon chilling acclimation, also exhibited better cold tolerance than wild-type controls, but were phenotypically normal. It should be noted that improvement of plant cold tolerance in the miR319 over-expression lines was more significant than that in the OsPCF5 and OsTCP21 RNAi lines (Yang et al., 2013), most likely due to target function redundancy in the latter case. This is also a challenge when manipulating miRNA targets instead of miRNAs themselves for plant trait modification, because in some cases multiple targets have to be simultaneously down-regulated to achieve the same level of effect as over-expression of an individual miRNA gene. Guan et al. (2013) discovered a novel plant thermotolerance mechanism, especially for the protection of reproductive organs. It involves induction of miR398 to downregulate its targets CSD (copper/zinc superoxide dismutase) genes, *CSD1* and *CSD2* as well as *CCS* (a gene encoding copper chaperone for both *CSD1* and *CSD2*; Guan et al., 2013). They found that csd1, csd2, and ccs mutants displayed higher heat stress tolerance than wild-type plants associated with increased accumulation of heat stress transcription factors and heat shock proteins and reduced damage to flowers (Guan et al., 2013). These results strongly suggest that manipulating miR398 or its targets can be an applicable strategy to increase heat tolerance in crop species, especially in corn, which suffers damage to its reproductive tissues by prolonged periods of high summer temperatures.

#### Oxidative Stress Tolerance

Plants tend to accumulate reactive oxygen species (ROS) in response to environmental stimuli such as high intensity light, extreme temperatures, UV radiation, heavy metals, salinity, drought stresses, and mechanical stresses (Khraiwesh et al., 2012). Superoxide dismutases (SODs) in plants can detoxify superoxide radicals by converting them into molecular oxygen and hydrogen peroxide. Several studies have been conducted to improve plant stress tolerance by over-expression of superoxide dismutase (Cu/Zn-SODs) in transgenic plants which detoxify superoxide radicals (Tepperman and Dunsmuir, 1990; Pitcher et al., 1991; Gupta et al., 1993; Perl et al., 1993; Sunkar et al., 2006). However, in some of the studies, the transgenic plants exhibited minimal or no increase in stress tolerance (Tepperman and Dunsmuir, 1990; Pitcher et al., 1991). Sunkar et al. (2006) offered an improved strategy to solve this problem by over-expressing miR398-resistant form of *CSD2*, which led to increased tolerance to high intensity light, heavy metals and other oxidative stresses (**Table 1**; Sunkar et al., 2006). The exploration of interactions between miR398 and its targets (*CSD1* and *CSD2*) also provided a possible explanation to the previously failed attempts. The expression of the introduced SOD transgenes containing the miR398 target sites were negatively impacted by miR398-mediated gene regulation.

### Nutritional Improvement

#### Biofortification

Essential fatty acids have important role in maintaining heart health in human. RNAi technology has also been used successfully or modification of fatty acid composition of oil. Two key fatty acid desaturase genes encoding stearoyl-acyl-carrier protein Δ9-desaturase and oleoyl-phosphatidylcholine γ6-desaturase (Liu et al., 2002) targeted by RNAi for nutritional enhancement of cotton seed oil with high-oleic (HO) and high-stearic (HS) contents. Soybean oil flavor and stability can be improved and process of hydrogenation can be avoided by reducing the content of alpha-linolenic acid (18:3). Flores et al. (2008) have used hairpin RNA mediated RNAi strategy (glycinin promoterwas used for seed-specific silencing) to down-regulate omega-3 fatty acid desaturase [an enzyme converting linoleic acid (18:2) to alpha-linolenic acid (18:3)]. Transgenic soybean seed were reported to significant reduction in alpha-linoleic acid content (1–3%) compared to non-transgenic soybean seed (7–10%).

Seed-specific RNAi approaches have also successfully been used to generate dominant high lysine corn by suppressing the expression of 22-kDa maize zein storage proteins, a group of abundant proteins in maize seed but poor in lysine content (Segal et al., 2003). RNAi was also used to down-regulate the starch-branching enzyme resulting in high-amylose wheat, which has a great potential to improve human health (Regina et al., 2006). RNAi technology can be applied to increase the starch content in the leaves. The process of phosphorylation and dephosphorylation of starch are crucial steps of leaf starch degradation. Based on this fact, starch content has been increased by manipulation of phosphate metabolism genes in *Arabidopsis* and maize (Weise et al., 2012). RNAi-mediated gene silencing has been utilized to reduce glutenin content in rice which is difficult to digest by kidney patients. To achieve low glutenin content GluB hairpin RNA was employed by Kusaba et al. (2003) to produce a rice variety called *LGC*-*1* (low glutenin content 1).

The two main enzymatic steps involved in sucrose biosynthesis involve synthesis of sucrose-6-phosphate (Suc6P) by sucrose-phosphate synthase (SPS) which is hydrolysed to sucrose and inorganic phosphate (Pi) by an another enzyme sucrose phosphatase (SPP). At low temperature (4^∘^C), potato tubers undergo an unfavorable phenomenon of, i.e., ‘cold sweetening’ which involves conversion of starch to reducing sugars glucose and fructose. To address this problem of cold induced sweetening, Chen et al. (2008) have targeted *NtSPP2* gene of tobacco for RNAi mediated suppression of SPP in transgenic potato tubers. The transgenic potato tubers showed accumulation of Suc6P when exposed to cold storage. In SPP-silenced tubers, the process of sucrose-to-hexose conversion was reduced as cold induced expression of vacuolar invertase (VI) was found to be blocked.

RNA interference technology was used by Gil-Humanes et al. (2008) to significantly reduce the level of γ-gliadins in different wheat cultivars by silencing the expression of specific γ-gliadins. Gil-Humanes et al. (2012) further, reported slight increase in the protein content of transgenic lines due to compensatory effect produced by down-regulation of γ-gliadins on the rest of gluten proteins. The glutenin content was found to increase whereas no significant changes were observed in the total gliadins content.

#### Allergen and Anti-Nutrient Elimination

Certain food like peanuts, apple, etc. consists of certain allergens that lead to allergic response when consumed. Besides many commonly consumed plants consist of natural toxin which is harmful to human health so, reduction or elimination of these allergens or toxins from food by RNAi will enhance its edibility and food quality. Expression of one of the major apple allergen Mald1 was reduced significantly by Gilisen et al. (2005) through RNAi-based gene silencing. RNAi was used by Le et al. (2006) to suppress the expression of a tomato allergen-Lyce3 which is a non-specific lipid transfer protein from tomato peel. Dodo et al. (2008) reduced the content of Arah2, a major peanut allergen by 25% in crude peanut extract. In onion, Eady et al. (2008) using hp RNAi suppressed the tear inducing lachrymatory factor synthase (LFS) gene which is involved in conversion of 1-propenyl sulfenic acid to propanthial *S*-oxide [tear-inducing, lachrymatory factor (LF)] resulting in the production of tearless onion. The RNAi technology has also been used to reduce the level of, a neurotoxin β-*N*-oxalyl-amino-alanine-L-alanine (BOAA) in the Grass pea. (*Lathyrus sativus*) or chickling pea which can cause paralytic disease called lathyrism (Angaji et al., 2010). Though cotton seeds are rich source of dietary protein but consist of a toxic terpenoid-gossypol which plays an important role in plant defense mechanism. RNAi has been used to lower gossypol content in cotton seeds by reducing seed specific expression of delta-cadinene synthase, an enzyme in gossypol biosynthesis pathway (Sunilkumar et al., 2006). Recently, Rathore et al. (2012) reported that RNAi-mediated silencing of delta-cadinene synthase gene resulted in cotton plants that produced ultra-low gossypol cottonseed (ULGCS). Lewis et al. (2008) used RNAi for silencing the nicotine demethylase gene to suppress the conversion of nicotine to nor-nicotine by six fold which is precursor of a carcinogenic compound in tobacco (*N. tabacum*).

## Conclusion and Future Prospects

RNA interference technology involving siRNA and miRNA have emerged as an attractive tool used by plant biologists not only to decipher the plant function but also to develop plants with improved and novel traits by manipulation of both desirable and undesirable genes. It is a powerful tool to understand the functions of individual genes and also proved useful to molecular breeders in producing improved crop varieties. sRNAs have been utilized either for silencing a gene of interest by RNAi or altering the expression of a gene by over-expressing a miRNA or introduction of artificially synthesized miRNA targeting it. The technology has well been utilized in crops not only to improve the food productivity (biomass and grain yield) but its nutritional value (cereals, fruits, and vegetables enriched with essential minerals, vitamins, fatty acids, and amino acids). RNAi technology has also been exploited to develop plants with improved resistance against various environmental stresses (especially drought) and biotic stresses like pathogen attack (virus, bacteria, insects, and nematodes).

Thus applications of research related with small non-coding RNAs has the potential to contribute to food safety by generating crop cultivars with improved agronomic traits leading to increasing yield and nutritional value. Research focussing on interaction of miRNAs and their targets can provides valuable insight into mechanisms of post-transcriptional gene regulation and multiple molecular pathways controlling plant stress responses. After completion of several recent genome sequencing projects, the study of RNAi has become increasingly more expanding and challenging. Researchers can manipulate several newly explored RNAi pathways in order to amend gene expression in crops, which can result in new and better quality traits.

Although RNAi technology can serve as potential tools for crop improvement but certain limitations are also associated with it. Altering the expression of a target gene or its miRNA might lead to undesirable pleiotropic changes in plant morphology and development. Therefore transgenic strategies should be designed only after completely understanding the mechanism of miRNA regulation. This will minimize undesirable trade-off in transgenic products. MiRNA strategy useful for one plant species may not work for another species, since regulations of evolutionarily conserved miRNAs may vary in different species (Li et al., 2008a; Zhang et al., 2011a). A family of related genes can be silenced with only one RNAi construct due to the sequence heterogeneity of siRNAs, but can cause off-target effects leading to unintentional silencing of genes with regions of homology to the intended target. Besides, post-transcriptional silencing in plants is mobile that can be induced locally and then spreads throughout the plant. Thus, siRNA-based RNAi strategies might not be suitable for some applications requiring tissue-specific silencing of genes. Atificial miRNAs can be used to overcome these limitations as it brings about much more specific silencing of genes. There is a reduced chance of off-target effects with miRNA-based RNAi, since miRNAs use only a single 21- or 22-nt sequence to identify the target, whereas siRNAs comprise a population of sequences. Thus, with miRNA-based RNAi, it is easier to target individual genes, even in a closely related gene family. amiRNAs are also suitable for tissue-specific silencing because miRNA-directed silencing does not spread throughout the plant. However, silencing might not be very durable with amiRNA because only a single 21- or 22-nt specificity determinant is involved. Mutation of the target can lead to easier escape from miRNA-directed silencing compared to siRNA-directed silencing which targets much larger sequence. This problem can be overcome by using two (or more) different amiRNAs against the target.

Crops engineered with constructs encoding heterologous proteins or over-expressing protein are fundamentally different from those consisting of RNA-mediated gene suppression cassettes because these constructs do not encode any protein(s) and are intended to express only non-coding RNAs. Transgenic proteins are recommended to undergo digestibility studies, to evaluate their potential for digestion, as allergenic and toxic proteins may be refractory to digestion (Astwood et al., 1996). RNA is not known to produce oral toxicity in humans but proteins in some rare cases can produce oral toxicity. According to the US FDA, “Introduced nucleic acids [in biotech crops], in and of themselves, do not raise safety concerns (FDA, 1992).” Furthermore, with regards to RNA-mediated gene regulation in genetically engineered crops, the US FDA states, “thus, for example, the introduction of a gene encoding an anti-sense ribonucleic acid (RNA) would not raise concerns about either the gene or the anti-sense RNA. Any safety considerations would focus on the intended effects of the anti-sense RNA.” Thus, transgenic crops with introduced RNA based traits are much safe for human consumptions than crops over-expressing proteins and need not require acute oral toxicity studies and evaluation of digestibility of the introduced RNA component. Certain bio safety concerns do arise on use of RNAi transgenic as transcriptional gene silencing by chromatin modification might lead to hereditary changes associated with adverse effects. However, crop plants developed with thoughtfully designed RNAi strategy and proper assessment of risk related to food safety may help overcome the biosafety concerns. In spite of few limitations, crop improvement strategies based on small non-coding RNAs have enormous potential to increase productivity as well as nutritional value.

## Conflict of Interest Statement

The authors declare that the research was conducted in the absence of any commercial or financial relationships that could be construed as a potential conflict of interest.
